# Beyond the Cocktail Effect: Pseudocapacitive Kinetics and Cycling-Induced Activation in a Ten-Cation High-Entropy Oxide Anode

**DOI:** 10.3390/nano16211344

**Published:** 2026-10-23

**Authors:** Yuseong Kim, Byoung-Nam Park

**Affiliations:** 1 Department of Chemical and Biomolecular Engineering, Lehigh University, Bethlehem, PA 18015, USA; 2 Department of Materials Science and Engineering, Hongik University, 72-1, Sangsu-dong, Mapo-gu, Seoul 04066, Republic of Korea

**Keywords:** pseudocapacitive storage, high-entropy, oxide anode, Li battery

## Abstract

Building on a previously established metal–organic framework (MOF)-inspired deca-nary high-entropy oxide anode/alternating current electrophoretic deposition (AC-EPD) platform, this study elucidates the multication distribution, charge-storage kinetics, and long-term cycling advantage of a binder- and conductive-agent-free deca-nary high-entropy anode. The active material was synthesized using a metal–organic framework 74 (MOF-74)-inspired deca-metal solvothermal coordination approach. Although the product evolved into a crystalline multi-metal oxide derivative rather than retaining the MOF-74 topology, energy-dispersive X-ray spectroscopy (EDS) confirmed the atomic-level homogeneous distribution of ten distinct cations (Mg, Ca, Sr, Ba, Mn, Fe, Co, Ni, Zn, Cd). The additive-free AC-EPD approach maximized the active material density and demonstrated the intrinsic electrochemical robustness of the high-entropy structure. Consequently, the electrode exhibited exceptional reversibility and superior rate capability (recovering ~310 mAh g^−1^ after 5.0 C cycling). Kinetic analysis revealed a dominant pseudocapacitive contribution (87% capacitive at 1.0 mV s^−1^), facilitating rapid lithium-ion transport. This work highlights a synergistic strategy combining MOF-inspired synthesis and electrophoretic fabrication for advanced high-entropy electrodes.

## 1. Introduction

The increasing demand for electric vehicles and high-performance portable electronics has spurred intensive research into advanced anode materials for lithium-ion batteries (LIBs) that offer both high power density and long-term cyclability [1,2,3]. While transition metal oxides (TMOs) have shown promise due to their high theoretical capacities, they often suffer from severe volume expansion and sluggish kinetics during lithiation/delithiation processes, leading to rapid capacity degradation [4,5]. To overcome these limitations, the concept of high-entropy oxides (HEOs) or multi-metallic complex oxides has recently emerged as a breakthrough strategy [6,7,8]. By incorporating five or more distinct metal cations into a single crystal lattice, these materials benefit from entropy stabilization and a synergistic “cocktail effect,” which can effectively mitigate structural strain and enhance electrochemical reactivity [9,10]. However, achieving atomic-level homogeneity in such complex multi-component systems remains a formidable challenge, as conventional solid-state synthesis methods frequently result in phase separation or inhomogeneous elemental distribution.

Following this concept, pioneering work by Sarkar et al. demonstrated the potential of a rock-salt-type high-entropy oxide, (Mg,Co,Ni,Cu,Zn)O, as a high-capacity anode for LIBs. They revealed that the entropy-stabilized crystal structure played a crucial role in maintaining reversible lithiation/delithiation processes, significantly outperforming individual metal oxides in terms of cycling stability [11]. Subsequently, the field expanded to include various crystal structures, such as high-entropy spinels [(Co,Cr,Fe,Mn,Ni)_3_O_4_] and perovskites, which consistently exhibited enhanced rate capabilities and suppressed particle agglomeration due to the robust multi-cation framework [12,13]. However, the majority of these reported high-entropy materials are synthesized via conventional solid-state reactions or high-energy ball milling. These top-down approaches often require excessive energy input and pose challenges in achieving atomic-level homogeneity, especially when the complexity of the system increases to accommodate ten or more distinct metal elements.

To fully capitalize on the intrinsic advantages of this high-entropy material, we employed alternating current electrophoretic deposition (AC-EPD) for electrode fabrication, departing from traditional slurry casting methods [14,15]. Conventional electrode preparation relies heavily on insulating polymer binders (e.g., poly(vinylidene fluoride) and conductive carbon additives, which inevitably reduce the gravimetric energy density by adding electrochemically inactive “dead weight” and can hinder efficient ion transport pathways. By utilizing AC-EPD, we successfully fabricated binder-free and conductive-agent-free electrodes, thereby maximizing the active material loading and ensuring direct electrical contact between the high-entropy oxide particles and the current collector. This additive-free configuration not only serves as a rigorous proof-of-concept for the material’s superior structural robustness and intrinsic electronic conductivity but also eliminates potential side reactions associated with binder decomposition. Furthermore, compared to direct current (DC) methods, the use of alternating current effectively suppresses solvent electrolysis and bubble formation, resulting in highly uniform and mechanically stable coatings essential for high-rate performance.

The deca-nary composition was selected based on the established M10M-MOF-74 framework, combining redox-active transition metals (Mn, Fe, Co, Ni) with alkaline-earth metals (Mg, Ca, Sr, Ba) and Zn/Cd to introduce diverse ionic radii and chemical environments [16]. This compositional design aims to promote multication redox synergy, lattice disorder, and potential structural buffering during repeated lithiation/delithiation. In our previous study, we established a proof-of-concept MOF-74-inspired route for preparing a deca-nary oxide and demonstrated its binder- and conductive-agent-free assembly by AC-EPD, together with its initial structural characterization and rate-capability assessment [17]. However, the kinetic origin of the favorable Li-storage behavior, the quantitative balance between diffusion- and surface-controlled processes, and the long-term advantage over a single-metal analogue remained unresolved. Building on this foundation, the present study combines EDS elemental mapping, scan-rate-dependent CV, *b*-value and Dunn analyses, Randles–Ševčík evaluation, and direct comparison with Fe-MOF-74 to elucidate how multication disorder promotes pseudocapacitive-dominant storage, rapid Li^+^ transport, and cycling-induced activation.

## 2. Materials and Methods

### 2.1. Preparation of Deca-Nary High-Entropy Oxide via MOF-Inspired Solvothermal Route

The precursor coordination chemistry was adapted from the M10M-MOF-74 protocol [16], whereas the conversion to a deca-nary high-entropy oxide anode (10M-HEO) and the binder-free AC-EPD electrode fabrication followed our previously reported procedure [17]. The principal experimental conditions are summarized below for reproducibility. Any modifications to the current collector, deposition conditions, or post-annealing treatment used in the present study are specified below. A deca-metal MOF-derived precursor was synthesized in a 150 mL glass vial by combining the organic linker H_4_DOT (300 mg) with a multication salt mixture comprising Mg(NO_3_)_2_·6H_2_O (116 mg), Ca(NO_3_)_2_·4H_2_O (106 mg), Sr(NO_3_)_2_ (96 mg), Ba(NO_3_)_2_ (118 mg), Mn(NO_3_)_2_·4H_2_O (113 mg), Fe(OAc)_2_ (79 mg), Co(NO_3_)_2_·6H_2_O (132 mg), Ni(NO_3_)_2_·6H_2_O (132 mg), Zn(NO_3_)_2_·6H_2_O (134 mg), and Cd(OAc)_2_·2H_2_O (120 mg). A mixed solvent system of dimethylformamide (DMF, 100 mL), ethanol (7 mL), and deionized water (7 mL) was added, and the suspension was sonicated until the solids were fully dissolved or uniformly dispersed, ensuring homogeneous coordination of the linker with all metal species. The vial was then tightly sealed and heated on a hot plate at 105–120 °C for 22 h to promote solvothermal MOF-like assembly. After the reaction, the product was recovered by centrifugation and repeatedly washed to remove unreacted species and residual mother liquor. Specifically, the solid was rinsed with fresh DMF (50 mL per cycle) over 3 days with a total of six solvent exchanges (total DMF volume: 300 mL), followed by solvent exchange into anhydrous methanol (100 mL per cycle) over 4 days with four exchanges (total MeOH volume: 400 mL). During each exchange, the supernatant was carefully removed using a pipette to minimize loss of fine particles. The washed solid was dried under vacuum for 16 h to evacuate trapped solvents and then thermally activated in a vacuum oven using a stepwise protocol—heating to 80 °C and holding for 1 h, then 100 °C for 1 h, 150 °C for 1 h, and finally 200 °C for 16 h—thereby ensuring thorough removal of coordinated/entrapped solvent prior to high-temperature conversion. To obtain the final multimetal oxide derivative, the activated precursor was calcined in a tube furnace under an argon atmosphere. The temperature was increased at 5 °C min^−1^ up to 700 °C and maintained at 700 °C for 1 h, yielding a crystalline oxide powder. The electrode is hereafter denoted as the 10M-HEO anode, where 10M-HEO refers to the MOF-inspired deca-nary high-entropy oxide containing Mg, Ca, Sr, Ba, Mn, Fe, Co, Ni, Zn, and Cd.

### 2.2. Binder-Free Electrode Fabrication via AC-EPD

For electrode preparation, 44 mg of the calcined powder was dispersed in ethanol to a concentration of 2 mg mL^−1^ (i.e., 22 mL total dispersion volume) and homogenized to form a stable suspension suitable for electrophoretic deposition. The suspension was then deposited onto 25 µm-thick stainless steel (SS) foil using AC electrophoretic deposition at 200 V and 3 Hz for 1 min, producing a typical mass loading of approximately 0.4–1.6 mg depending on deposition behavior. After deposition, the electrodes were subjected to a mild post-annealing step in an air furnace to improve interparticle contact and remove residual organics/adsorbates: the temperature was ramped at 3 °C min^−1^ to 200 °C and held for 1 h under air. This two-stage thermal processing (Ar calcination followed by low-temperature air treatment) enabled formation of the active oxide while maintaining a mechanically robust, additive-free electrode architecture on the Cu current collector.

### 2.3. Electrochemical Cell Assembly and Testing

CR2032 Li half-cells were assembled inside an Ar-filled glovebox, using Li metal as both the counter and reference electrode. Additive-free working electrodes were prepared by AC-EPD on stainless-steel (SS) foil (areal loading: 0.4 mg cm^−2^) without any binder or conductive carbon. The electrolyte was 1.0 M LiPF_6_ (Sigma-Aldrich, St. Louis, MO, USA) in an ethylene carbonate/diethyl carbonate mixture (EC/DEC = 1:1, *v*/*v*). After sealing, the cells were rested for 24 h in the glovebox to promote electrolyte wetting and interfacial stabilization. Electrochemical performance was evaluated using a battery test system. Galvanostatic charge–discharge (GCD) measurements were carried out between 0.01 and 3.0 V (vs Li/Li^+^) to assess rate capability over multiple C-rates. Cyclic voltammetry (CV) was performed at scan rates of 0.1–1.0 mV s^−1^ to probe redox characteristics and reaction kinetics.

## 3. Results and Discussion

**Figure 1:**
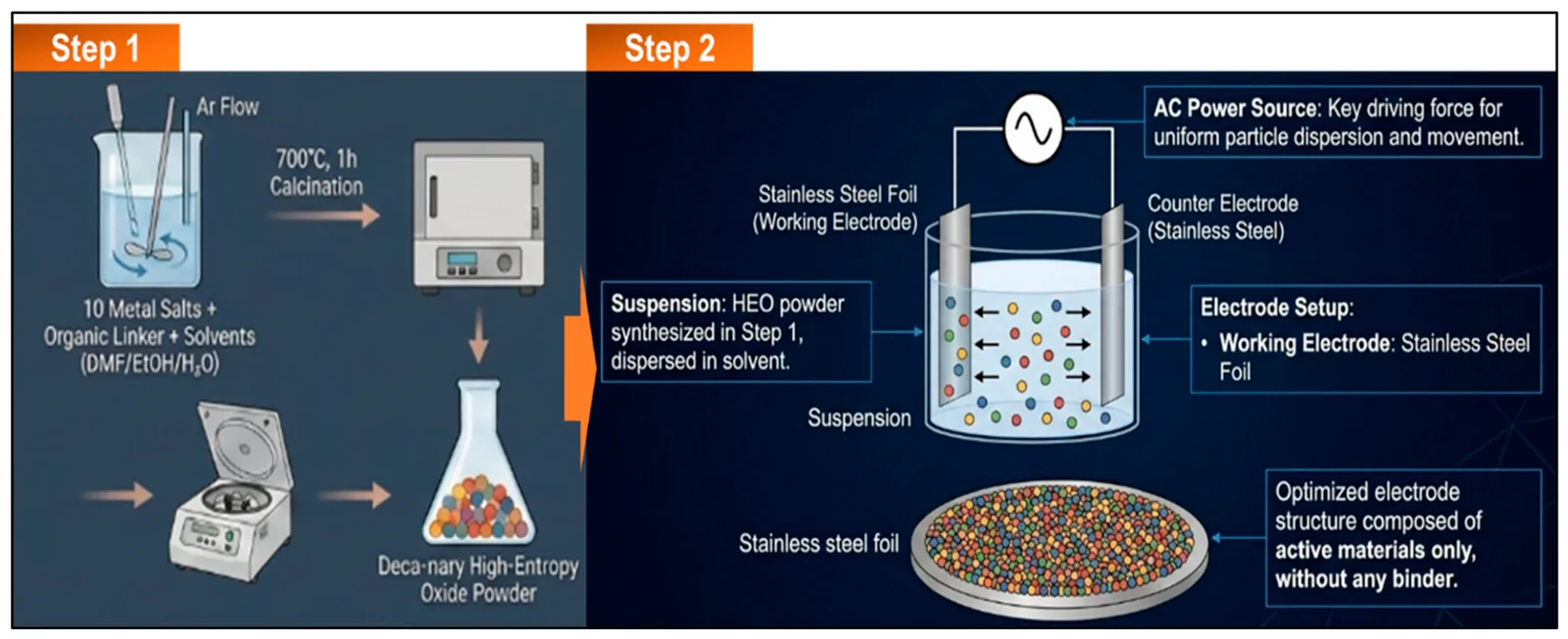
Schematic illustration of the two-step fabrication process for the binder-free 10M-HEO electrode. Step 1: A deca-nary high-entropy oxide powder is synthesized from ten metal salts, an organic linker, and mixed solvents, followed by Ar-assisted calcination at 700 °C for 1 h. Step 2: The obtained HEO powder is dispersed in solvent and directly deposited onto stainless steel foil by AC-EPD, producing an additive-free electrode composed only of active material without polymer binder or conductive carbon additives.

The synthesis feasibility, wurtzite-dominant structure, and initial rate response of the 10M-HEO electrode were established in our previous report [17]. The present study focuses on previously unresolved aspects, including multi-element spatial homogeneity, quantitative Li-storage kinetics, extended cycling behavior, and direct comparison with the single-metal Fe-MOF-74 reference. Figure 1 outlines our two-step strategy designed to maximize both the compositional entropy and the energy density of the 10M-HEO anode. The first step involves a MOF-inspired solvothermal synthesis, which is crucial for overcoming the thermodynamic immiscibility often encountered in multi-metallic systems. By utilizing the coordination chemistry of MOF precursors, we achieved an atomic-level mixing of ten different metal elements into a single high-entropy oxide phase, as will be demonstrated later. The second step highlights our electrode fabrication method via binder-free AC-EPD. Unlike conventional slurry casting, which relies on insulating binders and conductive agents that constitute electrochemical “dead weight,” our AC-EPD approach allows for the direct deposition of active materials onto the current collector. This additive-free configuration, as illustrated in the right panel of Figure 1, not only significantly enhances the volumetric and gravimetric energy density but also ensures robust electrical contact between the active particles and the substrate, thereby facilitating efficient electron transport during high-rate cycling.

Compared to conventional direct current electrophoretic deposition (DC-EPD), the alternating current (AC-EPD) technique offers distinct advantages, particularly for fabricating high-quality binder-free electrodes. The primary benefit of applying an AC field is the effective suppression of solvent electrolysis and the consequent minimization of gas bubble evolution at the electrode interface. In DC-EPD, gas generation often leads to porous, non-uniform coatings with poor adhesion and creates “dead” spaces within the electrode. In contrast, AC-EPD mitigates these electrochemical side reactions, enabling the formation of a highly dense, pinhole-free, and uniform film. This dense packing is critical for binder-free architectures, as it ensures robust mechanical integrity and intimate electrical contact between the high-entropy oxide particles and the current collector without the need for polymeric additives. Furthermore, AC-EPD prevents local pH fluctuations near the electrode surface, thereby preserving the chemical stability of the multi-metallic active material during the deposition process.

**Figure 2:**
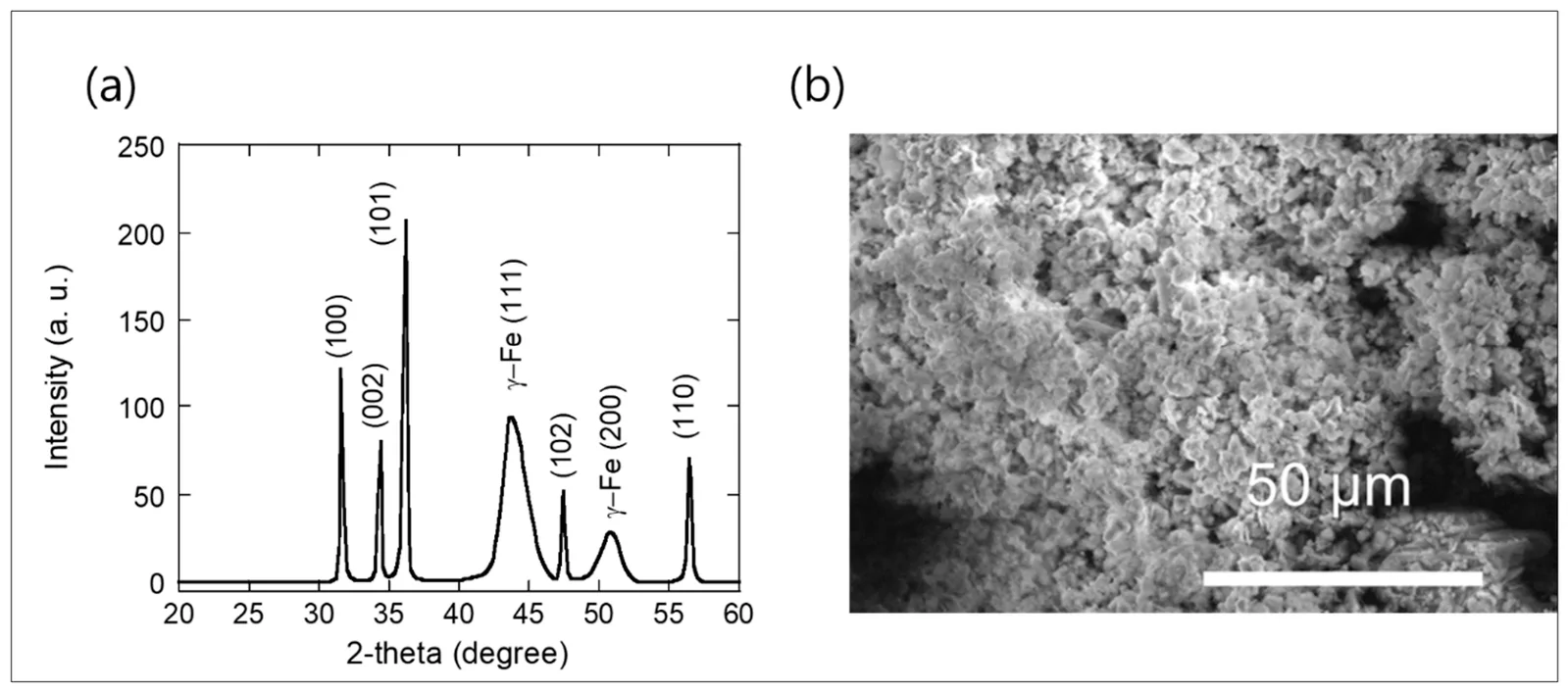
(**a**) X-ray diffraction of the 10M-HEO anode (after annealing) and (**b**) SEM image of the annealed sample.

X-ray diffraction (XRD) of the electrophoretically deposited 10M-HEO anode was employed to evaluate the crystalline phases formed after reacting the multi-cation precursor mixture (Ni, Zn, Mn, Fe, Co, Mg, Cd, Sr, Ca and Ba) in Figure 2a. The XRD pattern is dominated by reflections of the wurtzite ZnO structure, where the major peaks can be indexed to the (100), (002), (101), (102), and (110) planes [18]. Notably, two additional peaks appearing near 2θ ≈ 44° and ≈51° are assigned to the γ-Fe (austenite) reflections of the SS substrate/current collector (e.g., (111) and (200) of AISI 304/304L), rather than to the active material [19]. After accounting for these substrate-derived peaks, no other distinct reflections are observed within the measured range, supporting that the annealed product is primarily a ZnO-type solid-solution phase (i.e., a wurtzite host lattice accommodating multiple cations) rather than a mixture of separated oxide phases.

Notably, the ZnO-related reflections exhibit a small but systematic shift toward lower 2θ relative to typical reference positions for undoped ZnO. Such a coherent shift across multiple ZnO reflections implies a slight expansion of the ZnO lattice and is qualitatively consistent with partial incorporation of larger-radius cations into the ZnO framework (i.e., substitutional solid-solution formation) and/or the presence of residual microstrain. Using the fitted ZnO peak positions, the lattice parameters estimated for the wurtzite phase are a ≈ 3.256 Å and c ≈ 5.213 Å, indicating a modest expansion compared with commonly reported values for pristine ZnO. While this trend supports the ZnO-based solid-solution scenario, a definitive attribution of the peak shift to compositional effects requires correction for instrumental zero-shift (e.g., by using an internal standard) and quantitative refinement. Importantly, the absence of a complete set of accompanying reflections expected for common spinel ferrites or elemental Ni/Fe within the scanned range suggests that, if present, such phases are likely minor, strongly textured, or partially masked by background/substrate contributions.

**Figure 3:**
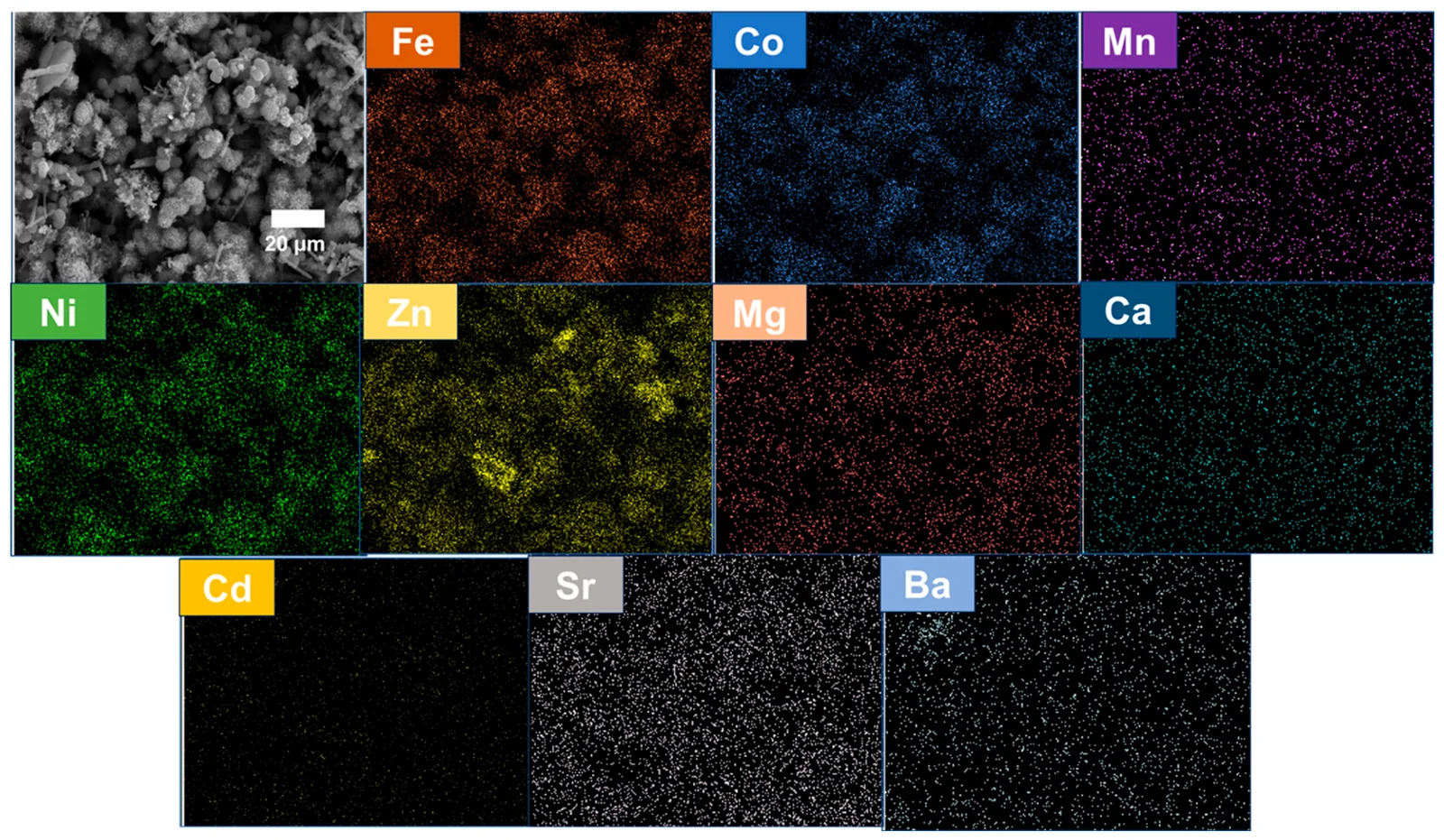
EDS mapping of the 10M-HEO anode deposited on an SS current collector.

To verify the successful incorporation and spatial arrangement of the deca-nary metal components, energy-dispersive X-ray spectroscopy (EDS) elemental mapping was conducted on the binder-free electrode surface. As presented in Figure 3, the mapping results reveal a remarkably uniform distribution of all ten metal elements—Mg, Ca, Sr, Ba, Mn, Fe, Co, Ni, Zn, and Cd—across the densely packed particles. The individual elemental maps exhibit consistent signal intensities that perfectly overlap with the morphological features observed in the SEM image, confirming that the ten distinct cations are homogeneously integrated throughout the material rather than existing as a physical mixture of separate metal oxides. Notably, transition metals such as Fe, Co, and Ni, which serve as primary redox-active centers, are well-dispersed without any localized agglomeration, while Zn and alkaline earth metals (Mg, Ca, Sr, Ba) form a consistent matrix skeleton. Although the ten metal precursors were introduced at approximately equimolar ratios (10 at% each), quantitative EDS analysis revealed a non-equimolar composition, with Fe (~39 at%), Co (~14 at%), and Mg (~11 at%) as the major constituents. The corresponding elemental composition is summarized in Figure S1, complementing the EDS mapping results and supporting the spatial distribution of multiple metal species within the 10M-HEO electrode.

The physical significance of this atomic-level homogeneity is substantial, as it provides direct evidence that the MOF-inspired solvothermal strategy successfully overcame the thermodynamic immiscibility often encountered in multi-metallic systems. Unlike conventional solid-state mixing, which is prone to phase segregation, our synthesis route facilitated the formation of a true high-entropy architecture where ten elements with varying ionic radii are intimately mixed. This uniform dispersion is crucial for activating the synergistic “cocktail effect,” where the presence of multiple cations increases the configurational entropy, thereby stabilizing the crystal structure against the mechanical strain induced by repeated lithiation and delithiation. Furthermore, the homogeneous distribution of conductive and structurally stable elements creates a robust percolation network, which effectively buffers volume expansion and ensures sustained electrochemical reversibility even at high current densities.

**Figure 4:**
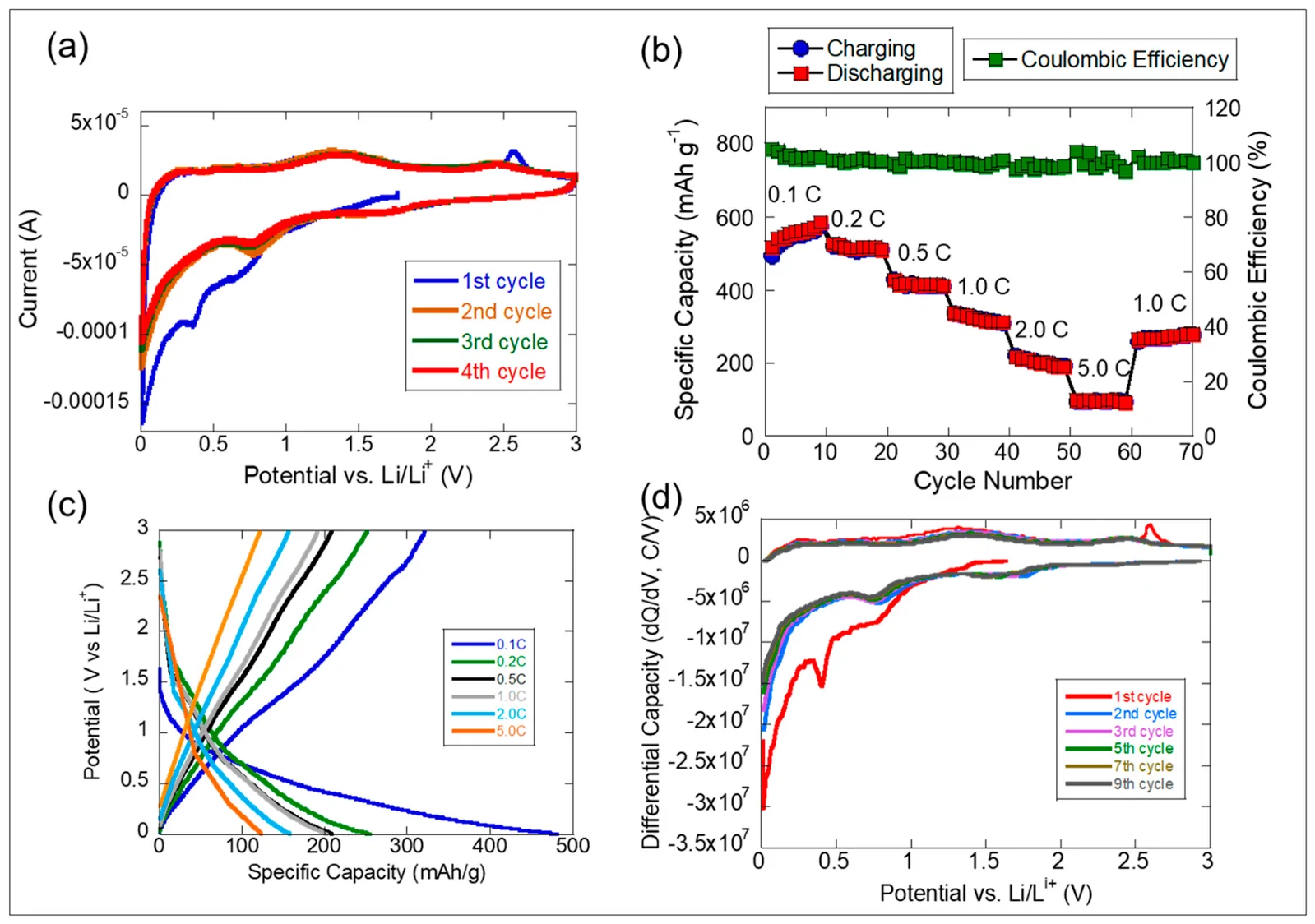
Electrochemical characterization of the 10M-HEO anode. (**a**) CV of the 10M-HEO electrode for the 1st–4th cycles, showing pronounced first-cycle irreversible reduction followed by highly overlapped subsequent cycles, indicating stabilized reversible Li^+^ storage. (**b**) Rate capability performance measured at stepwise current rates from 0.1 to 5.0 C and then returned to 1.0 C, together with coulombic efficiency. (**c**) GCD profiles at different C-rates, demonstrating preserved voltage profiles even under increased current density. (**d**) Differential capacity (dQ/dV) curves at selected cycles, highlighting the evolution of initial irreversible reactions and the subsequent stabilization of broad redox features during cycling.

To elucidate the Li storage mechanism of the 10M-HEO anode electrode, CV and differential capacity (dQ/dV) analyses were conducted. Figure 4a displays the CV curves at a scan rate of 0.1 mV s^−1^. In the first CV cycle, a pronounced cathodic peak appears around ~0.4–0.7 V vs. Li/Li^+^, corresponding to the initial reduction of the HEO anode into its metallic constituents (Ni, Co, Fe, Mn, etc.) with concurrent Li_2_O formation and solid–electrolyte interphase (SEI) deposition [20,21]. This initial conversion/SEI process is largely irreversible, as evidenced by the disappearance of the ~0.5 V peak in subsequent cycles [21]. During the first anodic sweep, a broad oxidation feature emerges around 1.2–2.5 V, attributable to the partial re-oxidation of the transition-metal species to higher oxidation states (e.g., Ni and Co oxidizing toward Ni^2+^/Ni^3+^ and Co^2+^/Co^3+^, and Fe^2+^/Fe^3+^) [20]. Owing to peak overlap from the many active metal cations, unambiguous assignment of each redox couple is difficult; instead, the CV profile reflects the combined redox activity of all components, including possible Ni^2+^/Ni^0^, Co^3+^/Co^2+^, Fe^3+^/Fe^2+^ and Mn^3+^/Mn^2+^ transitions. From the second cycle onward, the voltammograms overlap closely, indicating that most irreversible reactions occur only in the first cycle and that thereafter the electrochemical reactions are highly reversible [21]. The persistence of broad, stable redox peaks in later cycles also suggests a pseudocapacitive-like charge storage contribution, consistent with the improved cycle-to-cycle stability and reversible lithium storage behavior of the multicomponent oxide electrode.

In the rate capability test (Figure 4b), the electrode maintains stable capacities as the current density is stepwise increased from 0.1 to 5.0 C and then returned to 1.0 C. Even at a high current density of 5.0 C, the material retained a specific capacity of approximately 120 mAh g^−1^. More importantly, when the current density was returned to 1.0 C, the specific capacity instantly recovered to ~280 mAh g^−1^. Upon switching back to 1.0 C at ~60 cycles, the capacity is slightly lower than that obtained at ~30 cycles (the earlier 1.0 C segment), which can be reasonably attributed to a mild “rate-stress” effect—i.e., subtle interfacial reorganization such as SEI densification and a modest increase in charge-transfer resistance and/or partial loss of kinetic accessibility of some active sites after prolonged high-rate operation. Importantly, the fact that a substantial capacity is still recovered and remains stable at 1.0 C after the harsh 5.0 C cycling highlights the robust structural integrity and sustained efficiency remains close to 100% throughout the entire test, indicating highly reversible Li^+^ storage with minimal parasitic reactions (electrolyte decomposition, continuous SEI growth, or irreversible trapping of Li) and thus confirming that the electrode/electrolyte interface rapidly stabilizes and preserves near-quantitative charge balance during repeated cycling.

Figure 4c,d provide quantitative support for the trends observed in the CV and rate performance (Figure 4a,b). The galvanostatic voltage profiles (Figure 4c) exhibit predominantly sloping charge/discharge curves over 0–3 V vs. Li/Li^+^ with no extended flat plateau, consistent with the broad redox features in the CV (Figure 4a) and a significant surface/near-surface (pseudocapacitive-like) contribution, which will be discussed. The differential capacity plots (Figure 4d) further correlate with Figure 4a: the intense first-cycle cathodic feature centered near ~0.5 V (associated with the initial conversion/SEI formation) is strongly attenuated after the first cycle, whereas the remaining broad redox signatures (including oxidation contributions in the ~1.2–2.0 V region) overlap well from the 2nd cycle onward. This stabilization of dQ/dV profiles is consistent with the closely overlapping CV curves after activation (Figure 4a) and the near-unity coulombic efficiency during rate testing (Figure 4b), indicating that most irreversible reactions are confined to the initial cycle and subsequent cycling proceeds with highly reversible charge compensation.

**Figure 5:**
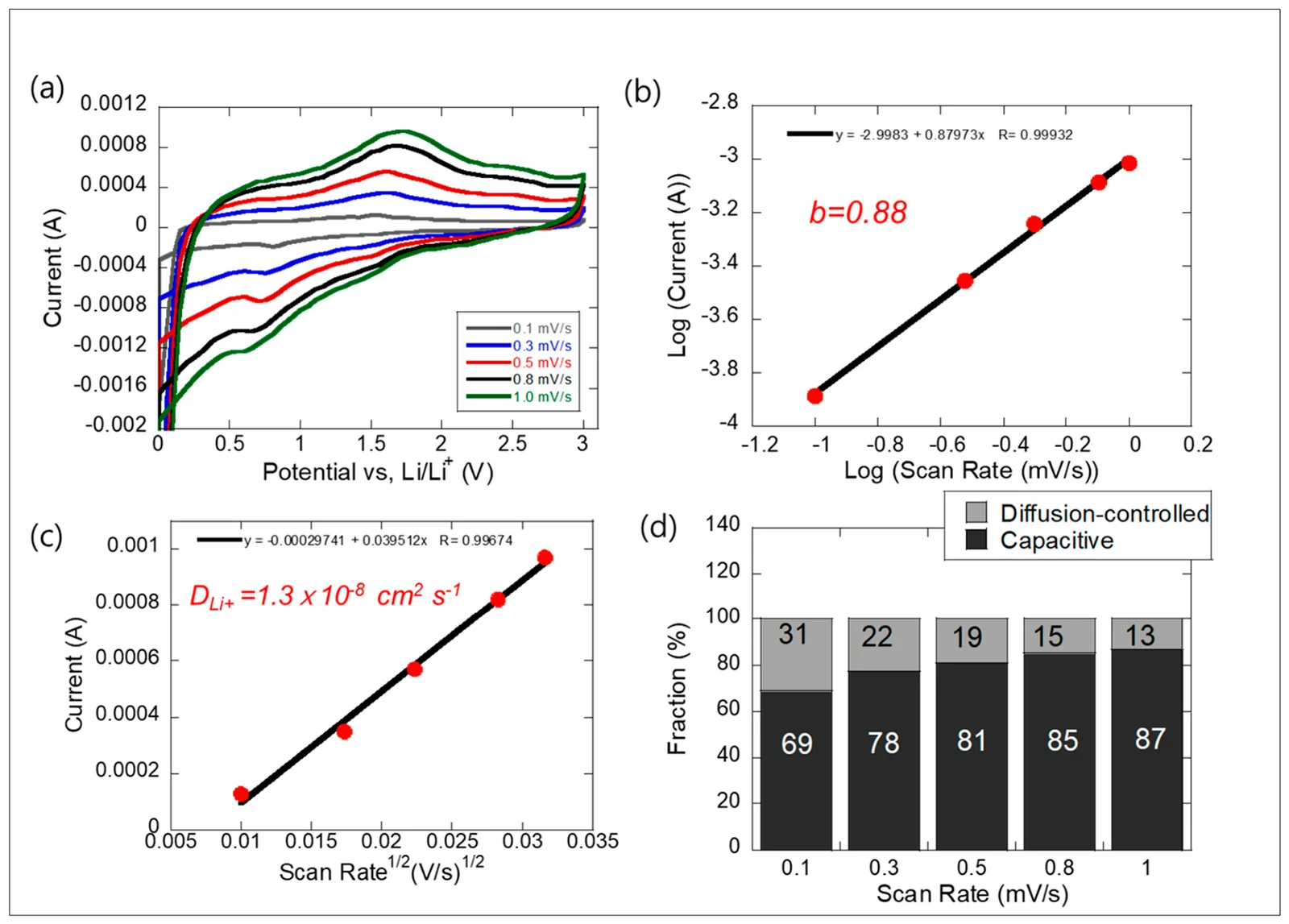
Kinetic analysis of Li^+^ storage in the 10M-HEO anode. (**a**) CV collected at scan rates of 0.1–1.0 mV s^−1^. (**b**) *b*-value analysis based on the relationship *i* = *aν^b^*, giving *b* = 0.88, indicative of dominant surface-controlled/pseudocapacitive kinetics. (**c**) Determination of the apparent Li^+^ diffusion coefficient from the linear relationship between peak current and the square root of scan rate using the Randles–Ševčík equation, yielding $D_{Li^{+}}\approx 1.3\times 10^{-8}$ cm^2^ s^−1^. (**d**) Dunn’s method analysis showing the capacitive and diffusion-controlled contributions at different scan rates. R represents the linear correlation coefficient (Pearson’s R) obtained from the linear regression analysis.

To further understand the origin of the excellent rate capability, the electrochemical kinetics of the M10M-derived electrode were investigated using CV at various scan rates (ν) ranging from 0.1 to 1.0 mV/s (Figure 5) to analyze charge-storage kinetics using the *b*-value and Dunn’s method. The *b*-value was obtained from the power-law relationship between peak current (*i*) and scan rate, *i* = *aν^b^*, where *b* ≈ 0.5 indicates diffusion-controlled (battery-type) behavior and *b* ≈ 1 suggests surface-controlled (capacitive/pseudocapacitive) kinetics. For quantitative separation of capacitive and diffusion contributions, Dunn’s approach expresses the current response at a given potential as *i*(*V*) = *k*_1_*ν* + *k*_2_*ν*^1/2^, where *k*_1_*ν* represents the surface-controlled (capacitive) component and *k*_2_*ν*^1/2^corresponds to the diffusion-limited contribution. By rearranging to *i*(*V*)/*ν*^1/2^ = *k*_1_*ν*^1/2^ + *k*_2_ and fitting *i*(*V*)/*ν*^1/2^ versus *ν*^1/2^at each potential, *k*_1_and *k*_2_ were extracted, enabling calculation of the capacitive fraction and diffusion fraction across the entire voltage window by integrating the corresponding currents [22].

As the scan rate increases in Figure 5, the CV shapes remain well-preserved with only subtle peak shifts, implying low polarization and efficient electron/ion transport within the electrode material. As shown in Figure 5b, the calculated *b*-value for the anodic peak is 0.88. Since a *b*-value of 0.5 indicates a diffusion-controlled process and a value of 1.0 represents a surface-controlled capacitive process, the obtained value of 0.88 suggests that the Li storage mechanism is dominated by surface-induced pseudocapacitance rather than slow bulk diffusion. This pseudocapacitive behavior is likely facilitated by the unique high-entropy surface structure, which provides abundant active sites for rapid Li adsorption and desorption.

To quantify the capacitive contribution to the total charge storage, the current response was separated into diffusion-controlled (*k*_2_*v*^1/2^) and capacitive (*k*_1_*v*) components. Figure 5d summarizes the contribution ratios at different scan rates. It is evident that the capacitive contribution increases with the scan rate, reaching a remarkable 87% at 1.0 mV/s. Even at a low scan rate of 0.1 mV s^−1^, the capacitive fraction remains dominant at 69%. This high ratio of capacitive charge storage confirms that the reaction kinetics are not limited by semi-infinite diffusion, enabling the rapid charge–discharge properties observed in the rate capability tests. The Li^+^ diffusion coefficient was estimated using the Randles–Ševčík equation from the linear relationship between peak current and the square root of scan rate (Figure 5c) [23], The apparent Li^+^ diffusion coefficient was calculated to be approximately 1.3 × 10^−8^cm^2^ s^−1^, indicating favorable Li^+^ transport kinetics in the high-entropy oxide electrode.

To further investigate the contribution of surface characteristics to Li-storage kinetics, Brunauer–Emmett–Teller analysis was performed, revealing a specific surface area of 97 m^2^ g^−1^ for the 10M-HEO material. This substantial surface area may enhance electrolyte accessibility and increase the number of electrochemically accessible surface/interfacial reaction sites, contributing to the high capacitive-like contribution of approximately 87% at 1.0 mV s^−1^. In addition, the chemically diverse multication architecture may facilitate distributed redox reactions and favorable charge-storage kinetics. Nevertheless, the respective contributions of surface area, morphology, and high-entropy composition cannot be independently quantified from the present results.

**Figure 6:**
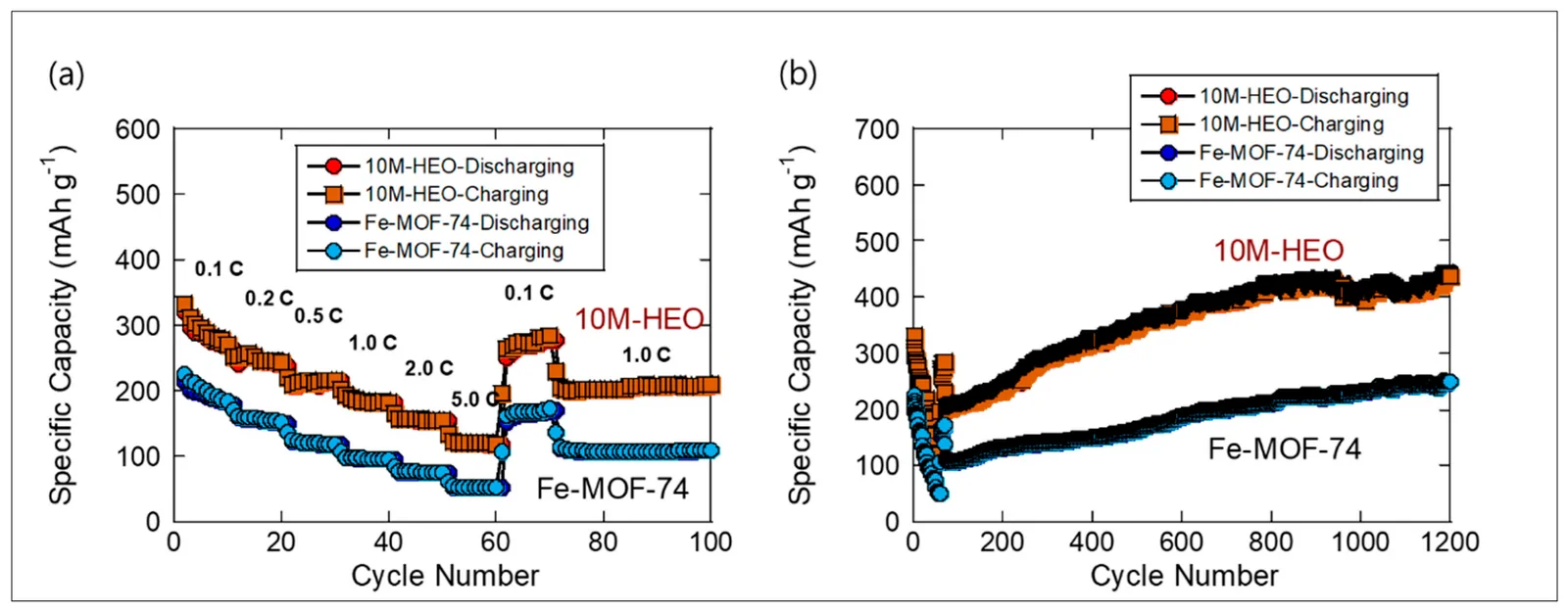
Comparative electrochemical performance of the 10M-HEO and Fe-MOF-74 electrodes. (**a**) Rate capability profiles measured at stepwise current rates from 0.1 to 5.0 C and then returned to 0.1/1.0 C, showing the higher reversible capacity of the 10M-HEO electrode across all rate conditions. (**b**) Long-term cycling performance (1C), where the 10M-HEO electrode exhibits a pronounced cycling-induced capacity increase and maintains substantially higher capacity than the Fe-MOF-74 reference electrode, highlighting the beneficial effect of the high-entropy multication architecture on long-cycle activation and charge-storage reversibility.

The electrochemical performance of the 10M-HEO anode was directly compared with that of its single-metal counterpart, the Fe-MOF-74-derived oxide. In the rate performance comparison (Figure 6a), the overall stability trends are similar for the 10M-HEO anode and Fe-MOF-74, suggesting that the binder-free electrode architecture ensures stable particle–current collector contact for both systems. In Figure 6b, both electrodes exhibit a capacity-increasing activation process typical of MOF-derived transition metal oxides; however, the magnitude of this enhancement is drastically different. The 10M-HEO anode shows a continuous and steep rise in capacity, stabilizing at a remarkably high value of ~450 mAh/g after 1200 cycles, which is nearly double the capacity exhibited by the Fe-MOF-74 (~250 mAh/g). In other words, the 10M-HEO anode maintains higher capacities at each rate step, while the long-cycling result reveals a more pronounced capacity rise with cycle number for the 10M-HEO anode. Although a definitive mechanistic conclusion requires additional impedance/operando analyses, the gradual increase is plausibly attributable to cycling-induced activation commonly reported for conversion-type oxide anodes (e.g., progressive electrode reorganization, improved percolation/charge-transfer pathways, and increased utilization of active sites). Therefore, the key distinction of the 10M-HEO anode in this comparison is not a dramatically different short-term rate stability, but a stronger long-term activation behavior that leads to higher accessible capacity during extended cycling.

The superior long-term cycling behavior of the 10M-HEO anode (Figure 6b) can be rationalized by a cycling-induced activation process that is frequently observed in conversion-type oxide anodes. During repeated lithiation/delithiation, conversion reactions generate and progressively reorganize a metal–Li_2_O nanocomposite (metallic nanoparticles embedded in a Li_2_O/oxide matrix), which can increase the electrochemically active interfacial area, open additional kinetically accessible sites, and gradually reduce polarization—manifesting as a steady capacity rise with cycle number. Importantly, the high-entropy, multi-cation configuration can further amplify this activation by suppressing phase segregation/coarsening and stabilizing the reaction microstructure under repetitive conversion stresses (i.e., entropy stabilization and “cocktail” synergy), thereby retaining a larger fraction of active interfaces over extended cycling. Recent mechanistic studies on high-entropy oxides also suggest that functional differentiation among elements can promote the formation of a self-organized nanostructure (e.g., an electronically percolating metallic network coupled to a stabilized oxide nanophase that accommodates Li^+^), which supports sustained charge transport and structural integrity even in micron-scale particles. Therefore, while the short-term rate stability may appear similar under the same binder-free electrode platform, the long-cycling data highlight the 10M-HEO anode’s key advantage: a more pronounced and persistent activation pathway that maintains (and progressively increases) the fraction of electrochemically utilized active sites during prolonged cycling.

The progressive capacity increase during prolonged cycling (Figure 6b) is consistent with cycling-induced activation reported for conversion-type high-entropy oxide anodes [24,25]. Repeated lithiation/delithiation may reorganize the reaction microstructure and increase the accessibility of Li-storage sites, while interconnected metal/oxide nanostructures, as demonstrated in a related HEO system, could facilitate charge transport [6]. However, interphase-related charge storage may also contribute to capacity growth. These processes have not been directly resolved in the present 10M-HEO electrode; therefore, the proposed activation mechanism should be regarded as an interpretation supported by the literature rather than conclusive evidence of structural evolution or entropy-driven stabilization.

In the single-metal Fe-MOF-74 system, the electrochemical reaction relies solely on the redox chemistry of iron, making the structure vulnerable to the severe volume changes and mechanical pulverization associated with repeated lithiation/delithiation. In contrast, the 10M-HEO anode incorporates a diverse mix of metals where active elements (e.g., Fe, Co, Ni, Mn) provide high capacity, while electrochemically stable elements (e.g., Mg, Zn, Ca) act as a “buffer matrix.” This configuration effectively dissipates mechanical stress and prevents the structural collapse that limits the lifespan of the Fe-MOF-74. Moreover, the severe lattice distortion characteristic of high-entropy oxides creates abundant defects and diffusion channels, facilitating the rapid ion transport observed in the rate capability tests. Consequently, the high-entropy strategy not only enhances the intrinsic structural integrity but also maximizes the utilization of active sites through a robust, self-reinforcing network that the single-metal system cannot achieve.

Compared with the Fe-MOF-74 electrode, which exhibits an apparent Li^+^ diffusion coefficient on the order of ~10^−10^ cm^2^ s^−1^, the 10M-HEO anode shows a much higher value of ~10^−8^ cm^2^ s^−1^, indicating substantially faster Li^+^ transport kinetics. This two-order-of-magnitude enhancement can be attributed to the unique high-entropy architecture. First, the homogeneous incorporation of multiple metal cations, confirmed by EDS elemental mapping, creates a chemically disordered and defect-rich lattice that can provide abundant Li^+^ transport pathways. The severe lattice distortion generated by the size and electronic mismatch among Mg, Ca, Sr, Ba, Mn, Fe, Co, Ni, Zn, and Cd may lower the kinetic barrier for Li^+^ migration by producing locally open and energetically diverse diffusion channels. Second, the coexistence of redox-active transition metals such as Fe, Co, Ni, and Mn can form spatially distributed reaction centers, while relatively stable cations such as Mg, Zn, Ca, Sr, and Ba help buffer structural stress during repeated lithiation/delithiation. This compositional synergy suppresses severe particle pulverization and maintains accessible ion/electron pathways during cycling.

In addition, the high apparent Li^+^ diffusivity is consistent with the dominant pseudocapacitive charge-storage behavior of the high-entropy electrode. Because a large fraction of the capacity is stored through fast surface/interfacial redox reactions rather than sluggish bulk diffusion, Li^+^ does not need to penetrate deeply into a single dense phase to participate in charge storage. Instead, the multication oxide provides numerous electrochemically accessible interfaces and defect sites, enabling rapid charge transfer and shortened Li^+^ diffusion lengths. Therefore, the higher diffusion coefficient of the 10M-HEO anode should be understood as an apparent kinetic enhancement arising from the combined effects of lattice disorder, multication redox activity, structural buffering, and pseudocapacitive-dominant Li^+^ storage. This explains why the 10M-HEO anode electrode delivers superior rate capability and stronger long-term activation compared with Fe-MOF-74.

**Figure 7:**
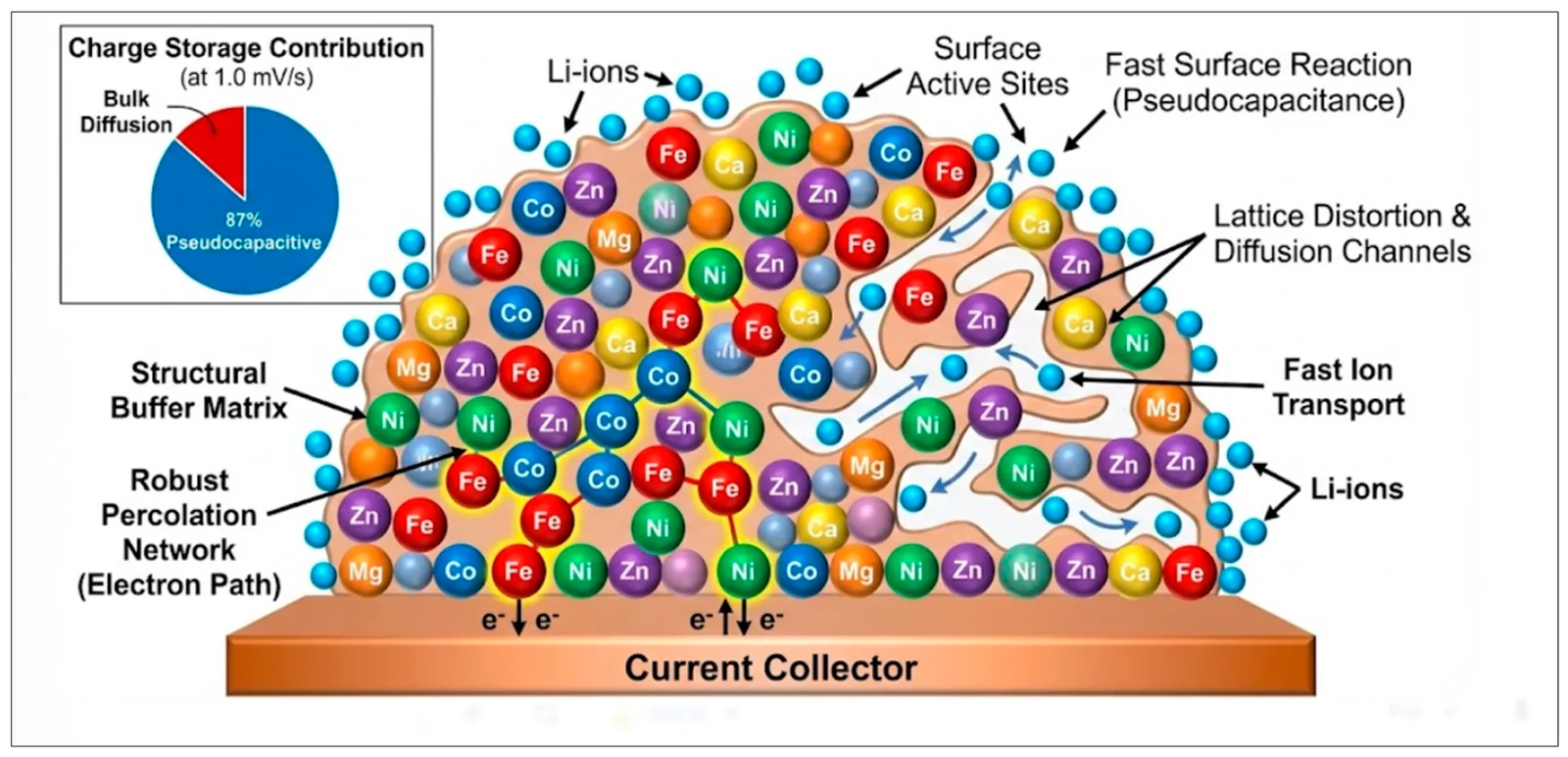
Schematic illustration of the proposed charge-storage mechanism in the 10M-HEO anode. The homogeneous multication architecture forms an integrated electron percolation network and structural buffer matrix, while lattice distortion and defect-rich diffusion channels facilitate rapid Li-ion transport. The abundant surface-active sites promote fast pseudocapacitive redox reactions, leading to a dominant pseudocapacitive contribution of ~87% at 1.0 mV s^−1^ and enhanced rate capability of the high-entropy electrode.

The superior electrochemical performance of the 10M-HEO anode is fundamentally driven by the synergistic interplay between a robust percolation network and a dominant pseudocapacitive charge storage mechanism as illustrated in Figure 7. The atomic-level integration of ten distinct metal elements establishes a resilient percolation network, where conductive species (e.g., Fe, Co, Ni) ensure continuous electron transport pathways, while electrochemically stable elements (e.g., Mg, Zn, Ca) act as a structural buffer to mitigate volume expansion during cycling. This structural integrity is further complemented by the severe lattice distortion inherent to the high-entropy architecture, which generates abundant defects and diffusion channels that serve as “superhighways” for rapid ion transport. Consequently, the charge storage process is primarily governed by surface-induced pseudocapacitance—accounting for approximately 87% of the total capacity at 1.0 mV/s—thereby enabling exceptional rate capability and long-term reversibility by bypassing the kinetic limitations of sluggish bulk diffusion.

## 4. Summary and Conclusion

In this work, we demonstrated a 10M-HEO anode prepared through a MOF-inspired solvothermal strategy combined with binder-free AC electrophoretic deposition. The key novelty of this study lies in the integration of ten metal species (Mg, Ca, Sr, Ba, Mn, Fe, Co, Ni, Zn, and Cd) into a compositionally homogeneous oxide framework, as supported by EDS elemental mapping and XRD analysis. The XRD pattern was dominated by wurtzite ZnO-type reflections with systematic peak shifts, indicating lattice expansion associated with multication incorporation, while a minor additional diffraction feature suggested only a small secondary or measurement-related contribution. This result highlights the effectiveness of the MOF-inspired coordination route for promoting homogeneous multication mixing beyond conventional solid-state or ball-milling approaches.

A second important contribution is the use of AC-EPD to fabricate binder- and conductive-agent-free electrodes. By eliminating electrochemically inactive additives, the electrode architecture allowed the intrinsic electrochemical behavior of the high-entropy oxide to be evaluated more directly while maximizing the fraction of active material. The high-entropy electrode exhibited superior rate capability and long-term cycling behavior compared with the Fe-MOF-74-derived reference electrode. In particular, the apparent Li^+^ diffusion coefficient increased from the order of ~10^−10^ cm^2^ s^−1^ for Fe-MOF-74 to ~10^−8^ cm^2^ s^−1^ for the high-entropy electrode, indicating substantially enhanced Li^+^ transport kinetics. This improvement is attributed to the combined effects of multication-induced lattice disorder, defect-rich diffusion pathways, spatially distributed redox centers, and structural buffering by electrochemically stable cations.

Furthermore, Dunn’s analysis revealed that the high-entropy electrode stores charge predominantly through fast surface/interfacial pseudocapacitive processes, with a capacitive contribution of approximately 87% at 1.0 mV s^−1^. This pseudocapacitive-dominant behavior explains the excellent rate performance and the progressive capacity activation observed during long-term cycling. Therefore, the high-entropy design not only increases compositional complexity but also creates a kinetically favorable and structurally resilient electrode environment.

Building on our previous proof-of-concept report of the 10M-HEO/AC-EPD platform, the present study advances this electrode from a performance demonstration to a mechanistic understanding of its fast and durable Li-storage behavior. EDS elemental mapping supports the spatially homogeneous distribution of the ten metal species, while scan-rate-dependent kinetic analyses reveal a dominant pseudocapacitive contribution. Direct comparison with Fe-MOF-74 further demonstrates that the principal advantage of the high-entropy architecture emerges during prolonged cycling through sustained cycling-induced activation, enhanced utilization of electrochemically accessible sites, and preservation of ion/electron transport pathways. These results establish multication disorder, pseudocapacitive-dominant storage, and structural buffering as the mechanistic basis for the long-term electrochemical advantage of the 10M-HEO anode.

## Data Availability

The data presented in this study are available on request from the corresponding author.

## Supplementary Materials

The following supporting information can be downloaded at https://www.mdpi.com/article/10.3390/nano16211344/s1, Section S1: Synthesis and Electrochemical Measurements; Figure S1: Quantitative EDS-derived elemental composition.

## References

[1] Zhang B., Wang X., Wang S., Li Y., Chen L., Jiao H., Yu Z., Tu J. (2025). High-energy-density lithium manganese iron phosphate for lithium-ion batteries: Progresses, challenges, and prospects. J. Energy Chem..

[2] Shahjalal M., Roy P.K., Shams T., Fly A., Chowdhury J.I., Ahmed M.R., Liu K. (2022). A review on second-life of Li-ion batteries: Prospects, challenges, and issues. Energy.

[3] Zhu Y., Xu X., Chen G., Zhong Y., Cai R., Li L., Shao Z. (2016). Surfactant-free self-assembly of reduced graphite oxide-MoO2 nanobelt composites used as electrode for lithium-ion batteries. Electrochim. Acta.

[4] Kim T., Song W., Son D.-Y., Ono L.K., Qi Y. (2019). Lithium-ion batteries: Outlook on present, future, and hybridized technologies. J. Mater. Chem. A.

[5] Zhao Y., Li X., Yan B., Xiong D., Li D., Lawes S., Sun X. (2016). Recent developments and understanding of novel mixed transition-metal oxides as anodes in lithium ion batteries. Adv. Energy Mater..

[6] Wang K., Hua W., Huang X., Stenzel D., Wang J., Ding Z., Cui Y., Wang Q., Ehrenberg H., Breitung B. (2023). Synergy of cations in high entropy oxide lithium ion battery anode. Nat. Commun..

[7] Zheng W., Liang G., Liu Q., Li J., Yuwono J.A., Zhang S., Peterson V.K., Guo Z. (2023). The promise of high-entropy materials for high-performance rechargeable Li-ion and Na-ion batteries. Joule.

[8] Zhao L., Lin Z., Zhong Y., Liu H., Sun X., Huang Y.-C., Rickard W.D., Tang T., Shao Z. (2025). Engineering of entropy-driven surface doping towards stabilized high-voltage NCM cathodes: Li (Ni, Co, Mn, Ce, La, Zr, Al) Ox. Mater. Rep. Energy.

[9] Chang C.-B., Lu Y.-R., Tuan H.-Y. (2023). High-entropy NaCl-type metal chalcogenides as K-ion storage materials: Role of the cocktail effect. Energy Storage Mater..

[10] Zhou Z., Ma Y., Brezesinski T., Breitung B., Wu Y., Ma Y. (2025). Improving upon rechargeable battery technologies: On the role of high-entropy effects. Energy Environ. Sci..

[11] Sarkar A., Velasco L., Wang D., Wang Q., Talasila G., de Biasi L., Kübel C., Brezesinski T., Bhattacharya S.S., Hahn H. (2018). High entropy oxides for reversible energy storage. Nat. Commun..

[12] Triolo C., Maisuradze M., Liu Y., Li M., Pagot G., Ponti A., Di Noto V., Aquilanti G., Pinna N., Giorgetti M. (2024). Role of the microstructure in the Li-storage performance of spinel-structured high-entropy (Mn, Fe, Co, Ni, Zn) oxide nanofibers. J. Electrochem. Soc..

[13] Ganesan S., Bhatt D., Satheesh D., Kokulnathan T., Pant P., Bharathi D.S., Baskar L., Sahoo N.G., Palaniappan A., Manavalan K. (2025). High entropy alloys: A comprehensive review of synthesis, properties, and characterization for electrochemical energy conversion and storage applications. J. Mater. Chem. A.

[14] Lee J.-Y., Park B.-N. (2026). Fabrication of additive-free manganese-1, 3, 5-benzenetricarboxylate thin-film anodes via alternating current electrophoretic deposition for stable lithium storage. Mater. Lett..

[15] Park B. (2025). Pseudocapacitive charge storage in ultrathin NiCo-metal organic framework-derived NiCo_2_O_4_ Li-metal batteries. Mater. Lett..

[16] Wang L.J., Deng H., Furukawa H., Gándara F., Cordova K.E., Peri D., Yaghi O.M. (2014). Synthesis and characterization of metal–organic framework-74 containing 2, 4, 6, 8, and 10 different metals. Inorg. Chem..

[17] Park B.-N. (2026). High-entropy Deca-nary (10-metal) oxide anodes derived from metal organic framework-inspired route for Li-metal batteries. Mater. Lett..

[18] Khorsand Zak A., Hashim A.M. (2025). Advanced XRD peak broadening analysis of gallium-doped ZnO nanoparticles for crystallite size evaluation. Sci. Rep..

[19] Tolchard J.R., Sømme A., Solberg J.K., Solheim K.G. (2015). On the measurement of austenite in supermartensitic stainless steel by X-ray diffraction. Mater. Charact..

[20] Shin D., Chae S., Park S., Seo B., Choi W. (2023). Rational engineering of high-entropy oxides for Li-ion battery anodes with finely tuned combustion syntheses. NPG Asia Mater..

[21] Sun X., Yan C., Chen Y., Si W., Deng J., Oswald S., Liu L., Schmidt O.G. (2014). Three-dimensionally “curved” NiO nanomembranes as ultrahigh rate capability anodes for Li-ion batteries with long cycle lifetimes. Energy Mater.

[22] Harish S., Sathyakam P.U. (2025). Dunn’s method for distinguishing charge storage mechanisms in supercapacitors: A status quo review. J. Electron. Mater..

[23] El-Latif E.I.A., Kebede M.A., Sekar K., Hameed T.A., Yahia I.S., Gao H., Sheha E. (2025). Modeling the diffusion coefficient of charge carriers in metal ion batteries using the Randles-Sevcik equation. Adv. Theory Simul..

[24] Ci N., Hu Y., Li Q., Cheng J., Zhang H., Li D., Li K., Reddy K.M., Ci L., Xie G. (2024). Cycling Reconstructed Hierarchical Nanoporous High-Entropy Oxides with Continuously Increasing Capacity for Li Storage. Small Methods.

[25] Ma G., Zheng Y., Meng F., Hu R. (2023). Understanding the lithiation mechanism of Li_2_O-doped spinel high-entropy oxides as anode materials for Li-ion batteries. Energy Adv..

