# Supplementary material for: Beyond the Cocktail Effect: Pseudocapacitive Kinetics and Cycling-Induced Activation in a Ten-Cation High-Entropy Oxide Anode
Source: Nanomaterials (Basel). 2026 Oct 23;16(21):1344. doi: 10.3390/nano16211344 (PMC13764875; doi:10.3390/nano16211344)

Supporting Information

Beyond the Cocktail Effect: Pseudocapacitive Kinetics and Cycling-Induced Activation in a Ten-Cation High-Entropy Oxide Anode

Yuseong Kim[1] and Byoung-Nam Park *[2]

[1]Department of Chemical and Biomolecular Engineering, Lehigh University, Bethlehem, Pennsylvania 18015, United States

[2]Department of Materials Science and Engineering Hongik University 72-1, Sangsu-dong, Mapo-gu, Seoul 04066, Korea;

*Correspondence: metalpbn@hongik.ac.kr

S1. Synthesis and Electrochemical Measurements

A deca-metal MOF-derived precursor was prepared in a 150 mL glass vial by combining the organic linker H_4DOT (300 mg) with $Mg(NO_3)_2·6H_2O$ (116 mg), $Ca(NO_3)_2·4H_2O$ (106 mg), $Sr(NO_3)_2$ (96 mg), $Ba(NO_3)_2$ (118 mg), $Mn(NO_3)_2·4H_2O$ (113 mg), $Fe(OAc)_2$ (79 mg), $Co(NO_3)_2·6H_2O$ (132 mg), $Ni(NO_3)_2·6H_2O$ (132 mg), $Zn(NO_3)_2·6H_2O$ (134 mg), and $Cd(OAc)_2·2H_2O$ (120 mg). DMF (100 mL), EtOH (6.7 mL), and H_2O (6.7 mL) were added, and the mixture was sonicated until the reagents were thoroughly dissolved/dispersed. The vial was sealed and heated on a hot plate at 105–120 °C for 22 h. After cooling, the resulting solid was collected by centrifugation and washed by solvent refreshment in DMF (50 mL per exchange; 6 exchanges over 3 days, for a total of 300 mL). The material was then solvent-exchanged with anhydrous MeOH (100 mL per exchange; 4 exchanges over 4 days, for a total of 400 mL), carefully decanting solvent with a pipette to minimize solid loss. Residual solvents were removed by evacuating in a vacuum oven for 16 h, followed by stepwise thermal activation under vacuum at 80 °C (1 h), 100 °C (1 h), 150 °C (1 h), and finally 200 °C (16 h).

The activated precursor was converted to the final oxide by calcination in a tube furnace under flowing Ar, ramping at 5 °C min^{-1} to 700 °C and holding for 1 h. For electrode fabrication, 44 mg of the calcined powder was dispersed in ethanol to a concentration of 2 mg mL^{-1} and deposited onto 25 μm Cu foil by AC electrophoretic deposition (200 V, 3 Hz, 1 min), yielding a typical loading of ~0.4–1.6 mg. After deposition, the electrodes were thermally treated in an air furnace by heating at 3 °C min^{-1} to 200 °C and holding for 1 h to complete the post-annealing step.

Secondary Phases and Additional Peaks

Besides ZnO, the pattern shows at least one additional crystalline component. In particular, there is a pronounced diffraction feature around 2θ≈43–44.6° that cannot be indexed to wurtzite ZnO. This appears as a broad peak centered near ~43.8–44.6° (Table 1). Several candidate phases could give rise to a reflection in this region: Rock-Salt-Type Oxides (MO, cubic)—many divalent metal monoxides crystallize in the NaCl-type (rock-salt) structure, which has a characteristic (200) reflection in the mid-40s 2θ. For example, NiO shows its (200) peak at 2θ ≈ 43.3°, and other $M^{2+}O$ like CoO, MgO, or (Mg,Co,Ni,Zn)O solid solutions exhibit a similar peak (the exact position depends on lattice constant a ~4.2–4.8 Å). A high-entropy solid solution oxide containing multiple 2+ cations in a single cubic phase—analogous to the known (Mg,Co,Ni,Cu,Zn)O rock-salt oxide—could produce a broad reflection near this angle. In our sample, the peak at ~44° is broad, suggesting poor crystallinity or overlapping contributions, consistent with a possible multi-cation oxide. If such a phase formed, it might incorporate smaller cations (Ni, Co, Mg, etc.) preferentially, yielding a slightly contracted lattice (higher angle) compared to pure NiO. (Indeed, the observed ~44.6° is a bit higher than NiO's 43.3°, implying a somewhat smaller cubic lattice parameter, possibly due to inclusion of small-radius ions like Mg^{2+}.) However, a definitive assignment is difficult—notably, a rock-salt phase would also show a (111) reflection near ~37°, which is not clearly observed (it would overlap with the strong ZnO 36.2° peak, but no distinct shoulder ~37.3° is evident). The (220) of such a cubic oxide (~62–63°) could coincide with ZnO's 62.9° peak. Given the data, a cubic (rock-salt) oxide solid solution is a plausible minor phase, but its presence is largely inferred from the single (200) peak around 44°.

Another possibility for a peak near 44° is a spinel-type oxide. Common spinel ferrites (e.g. $ZnFe_2O_4$, $NiFe_2O_4$, $CoFe_2O_4$, etc.) have a strong reflection around 2θ ≈ 35.2–35.6° ((311) plane) and another around 43–44° ((400) plane). For instance, cubic $NiFe_2O_4$ has peaks at 2θ ≈ 30.3°, 35.6°, 37.3°, 43.3°, 53.7°, 57.2°, 62.9° (indexed to (220), (311), (222), (400), (422), (511), (440) respectively). $ZnFe_2O_4$ similarly shows reflections at ~29.9°, 35.2°, 56.7°, 62.3° (among others). In our pattern, we do not see the hallmark spinel (311) peak at ~35.3° (it would lie just below the intense ZnO 36.2° peak, but there is no clear separate peak at ~35.5°). We also do not observe a distinct ~30° (220) peak above background. This makes a major spinel phase unlikely. If a spinel formed (for example, involving Fe^{3+} from partial oxidation of Fe^{2+}, combined with Zn/Co/Mn), it must be in a very low fraction or its peaks are obscured by ZnO. The lone ~43–44° feature could correspond to a spinel (400) reflection, but without the accompanying spinel peaks (311, 220, 511, etc.), a spinel phase cannot be definitively confirmed. We consider that at most a minor spinel ferrite might be present, given that Fe and other transition metals are in the system—e.g. a Zn–Fe–Co mixed ferrite could form as an impurity. Its absence in XRD (beyond possibly the 44° line) suggests any such spinel domains are either very small or overshadowed by ZnO.

Metallic nickel or iron could produce a diffraction around this angle. For example, Ni (face-centered cubic) has a primary (111) peak at 44.5° (Cu Kα), and α-Fe (body-centered cubic) has its (110)

peak at ~44.7°. A small amount of Ni or Fe metal (from incomplete oxidation or reduction during synthesis) could thus give a peak ~44.5°–44.7°. If Ni^0 or Fe^0 were present, one might also expect secondary metal peaks [Ni(200) at ~51.8°; Fe(200) at ~65°]. Our pattern shows a very weak bump around ~51° but nothing clear at 65°. This, plus the broadness of the ~44.5° feature, suggests metallic Ni/Fe, if present, is minor and likely nanocrystalline (broad peak).

Figure S1. Quantitative EDS-derived elemental composition of the 10M-HEO electrode, showing the relative atomic percentages of Mg, Ca, Sr, Ba, Mn, Fe, Co, Ni, Zn, and Cd, normalized to the total metal content.

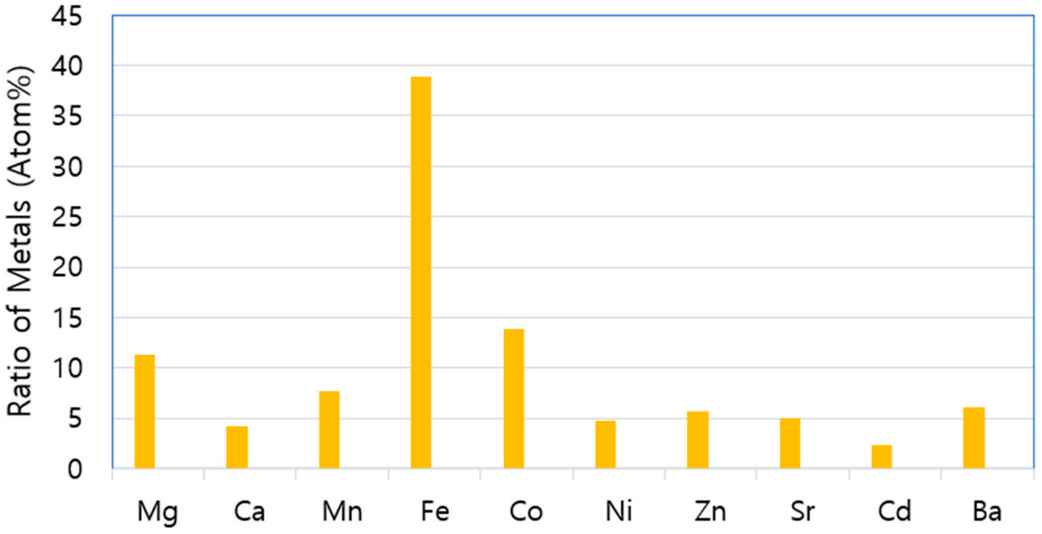

Supplement: Supplementary file 1 [file nanomaterials-16-01344-s001.zip › nanomaterials-4636995-supplementary.pdf]
